# Germline variants in hereditary breast cancer genes are associated with early age at diagnosis and family history in Guatemalan breast cancer

**DOI:** 10.1007/s10549-021-06305-5

**Published:** 2021-07-01

**Authors:** Megan Ren, Anali Orozco, Kang Shao, Anaseidy Albanez, Jeremy Ortiz, Boyang Cao, Lusheng Wang, Lilian Barreda, Christian S. Alvarez, Lisa Garland, Dongjing Wu, Charles C. Chung, Jiahui Wang, Megan Frone, Sergio Ralon, Victor Argueta, Roberto Orozco, Eduardo Gharzouzi, Michael Dean

**Affiliations:** 1 Division of Cancer Epidemiology and Genetics, National Cancer Institute, NIH, Gaithersburg, MD USA; 2 Instituto Cancerologia, Guatemala City, Guatemala; 3 BGI-Shenzhen, Beishan Industrial Zone, Shenzhen, 518083 People’s Republic of China; 4 City University of Hong Kong Shenzhen Research Institute, Shenzhen, People’s Republic of China; 5 Department of Computer Science, City University of Hong Kong, Kowloon, SAR, Hong Kong, People’s Republic of China; 6 Hospital General San Juan de Dios, Guatemala City, Guatemala; 7 Cancer Genetics Research Laboratory, Division of Cancer Epidemiology and Genetics, Frederick National Laboratory for Cancer Research, Gaithersburg, MD USA; 8 Integra Cancer Center, Guatemala City, Guatemala; 9 Present Address: Office of Biostatistics and Epidemiology (OBE), Center for Biologics Evaluation and Research (CBER), Food and Drug Administration (FDA), Silver Spring, MD 20993-0002 USA

**Keywords:** *BRCA1* gene, *BRCA2* gene, Health disparities, Latin America, Hispanic, Pathogenic mutation

## Abstract

**Purpose:**

Mutations in hereditary breast cancer genes play an important role in the risk for cancer.

**Methods:**

Cancer susceptibility genes were sequenced in 664 unselected breast cancer cases from Guatemala. Variants were annotated with ClinVar and VarSome.

**Results:**

A total of 73 out of 664 subjects (11%) had a pathogenic variant in a high or moderate penetrance gene. The most frequently mutated genes were *BRCA1* (37/664, 5.6%) followed by *BRCA2* (15/664, 2.3%), *PALB2* (5/664, 0.8%), and *TP53* (5/664, 0.8%). Pathogenic variants were also detected in the moderate penetrance genes *ATM*, *BARD1*, *CHEK2*, and *MSH6*. The high ratio of *BRCA1*/*BRCA2* mutations is due to two potential founder mutations: *BRCA1 c.212* + *1G* > *A* splice mutation (15 cases) and *BRCA1 c.799delT* (9 cases). Cases with pathogenic mutations had a significantly earlier age at diagnosis (45 vs 51 years, *P* < 0.001), are more likely to have had diagnosis before menopause, and a higher percentage had a relative with any cancer (51% vs 37%, *P* = 0.038) or breast cancer (33% vs 15%, *P* < 0.001).

**Conclusions:**

Hereditary breast cancer mutations were observed among Guatemalan women, and these women are more likely to have early age at diagnosis and family history of cancer. These data suggest the use of genetic testing in breast cancer patients and those at high risk as part of a strategy to reduce breast cancer mortality in Guatemala.

**Supplementary Information:**

The online version contains supplementary material available at 10.1007/s10549-021-06305-5.

## Introduction

Breast cancer is the most commonly diagnosed cancer among women worldwide. In Latin America and the Caribbean, breast cancer accounted for 15% of all cancer cases among women in 2018 [1]. Genetic testing for germline mutations in breast cancer susceptibility genes can identify individuals with a higher risk of developing breast cancer. Still there is limited information on the mutation profile of many Latin American populations [2, 3]. A study conducted in Latin American women referred for genetic testing showed that they have equal or higher rates of *BRCA1* and *BRCA2* mutations (15%) as other groups [4]. The frequency of hereditary cancer gene mutations has been described for other Latin American populations and U.S. Hispanics, but only one previous study included 19 patients from Guatemala[5–8].

The Guatemalan census completed in 2018 (https://www.censopoblacion.gt/cuantossomos) describes a population of 14.9 million, composed of 44% who self-identify as indigenous (nearly all from Mayan language groups) and 53% Mestizo (mixed European and Amerindian ancestry). Genetic analysis of Guatemalan populations found that Mayan individuals average 92% Amerindian and 8% European ancestry and Mestizos 55% Amerindian and 41% European [9]. Breast cancer in Guatemala accounts for 19% of female cancer cases [1]. Therefore, to understand breast cancer genetic susceptibility in the Americas, it is important to have a well powered study from Guatemala.

The *BRCA1* and *BRCA2* genes are highly penetrant susceptibility genes for hereditary breast cancer with pathogenic variants found in 5–10% of unselected subjects [10]. Recurrent mutations have been described, such as *BRCA1* 185delAG, *BRCA1* 5382insC, and *BRCA2* 6147delT that explain 78% of cases in Ashkenazi Jews [11]. In Latin America, variants such as *BRCA1* 185delAG and R1443X are among the 20 most frequent *BRCA1* variants reported by the Breast Cancer Information Core database. Others like *BRCA1* A1708E are among the 10 most frequent pathogenic variants in Latin America [3]. This study analyzes the breast cancer variant profile in Guatemala, where few genomic studies currently exist for breast cancer [6]. The cultural and genetic diversity of Latin America has been well described [3, 9, 12]. Therefore, understanding breast cancer genetic risk in specific populations is relevant for the development of effective local prevention strategies and the results from this study can inform clinical diagnostics for Guatemalan women both abroad and in the U.S.

## Methods

### Study design and data collection

This study was conducted at the Hospital General San Juan de Dios (HGSJDD) and the Instituto de Cancerología (INCAN) in Guatemala City. HGSJDD is the largest public general hospital in the country and is a referral center for all regional public hospitals. INCAN is the largest cancer hospital in Guatemala and is managed by a foundation, the Liga Nacional Contra el Cancer, but receives support from the government to treat patients referred through public hospitals. The Research Ethical Committees approved the protocol and declared the study exempt from institutional review board (IRB) approval by the NIH Office of Human Studies Research. Women gave written informed consent. Women over the age of 18 referred for a diagnostic biopsy of a breast mass were recruited into the study. Nearly all subjects had a palpable mass with involvement of axillary lymph nodes. There were no other inclusion criteria, and the only exclusion was for women unable or unwilling to provide informed consent. HGSJDD subjects were recruited from January 2017 to June 2019, and only biopsy confirmed invasive breast cancer cases included. INCAN patients were recruited from August 2014 to December 2017, and biopsy results were not available. The subjects were not consecutive patients, and we estimate that we captured 10–20% of women diagnosed with breast cancer at these centers.

#### Patient demographic and reproductive history information

To obtain patient demographic and reproductive history information, trained interviewers administered an approved questionnaire. The answers were entered into a relational database and checked against paper records.The questionnaire included, age, age of menarche, menopausal status, number of pregnancies and miscarriages, previous breast cancer diagnosis, smoking, oral contraception use, history of breast feeding, languages spoken, self-identified ethnicity, mammography history and estrogen therapy use.

### Sample preparation and whole-exome sequencing

 was extracted from peripheral blood samples by a Qiagen DNA Blood Mini kit (Qiagen, Hilden, Germany)and quantitated by a Qubit Fluorometer (Life Technologies).

### Next-generation sequencing and variant calling

Targeted sequencing was performed on cancer susceptibility genes, including *BRCA1, BRCA2, PALB2, PTEN, TP53, ATM, BARD1, BRIP1, CHEK2, MSH6, RAD51D, STK11* (Supplemental Tables 1, 2). Genes with a relative risk (RR) for breast cancer > 5 were designated High risk genes, and those with a RR > 1.5 as Moderate risk. A description of the genes along with their relative risk for breast cancer [13] can be found in Supplemental Table 1.

Gene target regions were captured, and DNA sequencing was performed at the National Cancer Institute and BGI Shenzhen. A total of 587 samples from INCAN were sequenced on the BGISEQ-500 platform (MGI, a BGI Company) with Paired-end 100 bp and 86 samples from HGSJDD were sequenced on a Hiseq 2500 (Illumina) with the Paired-end 200 bp strategy (Supplemental Table 2).

For the samples on the BGISEQ-500 platform, 1ug DNA was fragmented to 200–400 bp by Covaris E210 (Covaris Inc.). The coding region and boundaries of 115 genes were captured by a BGI capture array. The average depth was 650× (235 × minimum) with 99% coverage. Reads were filtered with SOAPnuke 1.5.0 and assembled with BWA 0.7.12. Bam files were processed with Samtools 1.2, and duplicates identified with MarkDuplicates 1.138. GATK 3.4 was used for alignment and germline mutations calling.

For the Hiseq platform, 1 ug genomic DNA was fragmented, and the library was constructed according to the manufacturer’s protocols to an average depth of 200X and over 99% coverage on target regions.

Variants were annotated using automated pipelines and potential pathogenic variants were identified. Further validation was performed by manual review using the Integrative Genomics Viewer (IGV) [14]. Variants were classified, and the pathogenicity analyzed using ClinVar and Varsome.

To determine genetic testing qualification, we used a modified National Comprehensive Cancer Network (NCCN) criteria. Briefly, NCCN recommends testing for breast cancer patients age 45 or younger; 46–50 years with unknown family history or one or more close relatives (defined as 1st, 2nd, or 3rd degree relatives) with breast, ovarian, or prostate cancer at any age; breast cancer diagnosed at any age with one or more close blood relatives with breast cancer age 50 or younger or ovarian, pancreatic, or metastatic of high-risk phenotype breast cancer at any age; or 3 or more total diagnoses of breast cancer in the individual or close blood relatives. (https://www.nccn.org/professionals/physician_gls/pdf/genetics_bop.pdf). Due to having only self-reported family history, we included all patients diagnosed under age 50.

### Statistical analysis

Patients with and without a pathogenic mutation in a high or moderate penetrance gene were compared for age at diagnosis, menopausal status and family history of cancer or breast cancer. Median and interquartile ranges [IQRs] were calculated for the continuous variables, while frequencies and percentages were computed for the categorical variables. Wilcoxon rank-sum test was used to examine differences between age at diagnosis, age at menarche, age at first pregnancy, number of children, number of pregnancies, with the presence of pathogenic mutations in SAS software v 9.4 (SAS Institute, Cary, NC). A complete case analysis approach was used in our analysis as overall missing rate of covariates was small. Also, a two proportion *Z*-test (two-tailed) was used to assess the difference in the percentage of patients having a family history of cancer, family history of breast cancer, contraception use, and NCCN status. In all calculations, a p-value of 0.05 or less was deemed significant.

## Results

### Study population

In total, 664 patients with breast cancer were recruited from the Instituto de Cancerología and Hospital General San Juan de Dios, in Guatemala City. Most patients self-identified as “Mestizo,” a category used in many Latin American countries to refer to people of mixed European and Indigenous American ancestry [9]. The median age at diagnosis of the breast cancer cases was 49 (IQR 41–61]) (Table 1). The median number of children per study participant was 3 [IQR 2–4], and the median age at menarche was 13 [IQR 12–14]. Furthermore, 57% of patients were postmenopausal at diagnosis. Of the patients with family history information, 17% had at least one first- or second-degree relative with breast cancer.

**Table 1:** Study population characteristics overall and by the presence of pathogenic mutations in high and moderate genes

	Overall	Pathogenic^1^	Others^2^	*P*-value^3^
	(*N* = 664)	(*n* = 73)	(*n* = 591)	
Age at diagnosis, median (IQR)^4^	49 (41, 61)	43 (37, 52)	50 (41, 61)	< 0.01
Age at first pregnancy, median (IQR)^4^	21 (18, 25)	20 (17, 25)	21 (18, 25)	0.40
Age at menarche, median (IQR)^4^	13 (12, 14)	13 (12, 14)	13 (12, 14)	0.33
Number of children, median (IQR)^4^	3 (2, 4)	3 (2, 4)	3 (2, 5)	0.24
Number of pregnancies, median (IQR)^4^	3 (2, 5)	3 (2, 4)	3 (2, 5)	0.11
Miscarriage ≥ 1^4^	86 (15.1)	4 (0.7)	82 (14.4)	0.08
Breastfed, *N* %^4^	521 (90.4)	57 (91.9)	465 (90.5)	0.71
Menopause, *N* %^4^	361 (57.1)	32 (43.4)	348 (58.8)	0.02
Family history of cancer, *N* %^4^	243 (38.4)	34 (50.7)	210 (37.2)	0.03
Family history of breast cancer, *N* %^4^	107 (16.9)	22 (32.8)	85 (15.0)	< 0.01
Contraception use, *N* %^4^	138 (21.8)	9 (13.4)	129 (22.8)	0.08
Smoking, *N* (%)^4^	36 (5.7)	3 (4.5)	31 (5.8)	0.68
NCCN, *N* (%)^4^	354 (62.8)	48 (78.7)	306 (60.8)	< 0.01

Demographic and reproductive characteristics are compared between subjects with pathogenic mutations in high and moderate penetrance genes and those with benign or no variants (other). The median and interquartile range (IQR) are shown along with *P*-values for the comparison between subjects with and without pathogenic mutations in high- and moderate-risk breast cancer genes (Wilcoxon Two-Sample Test)

^1^High and medium penetrance

^2^Other or no variants

^3^*P*-values calculated using the chi square test, Fisher's exact test, or Wilcoxon test

^4^Categories do not sum to the totals due to missing data

### Germline mutations in breast cancer susceptibility genes

**Fig. 1:**
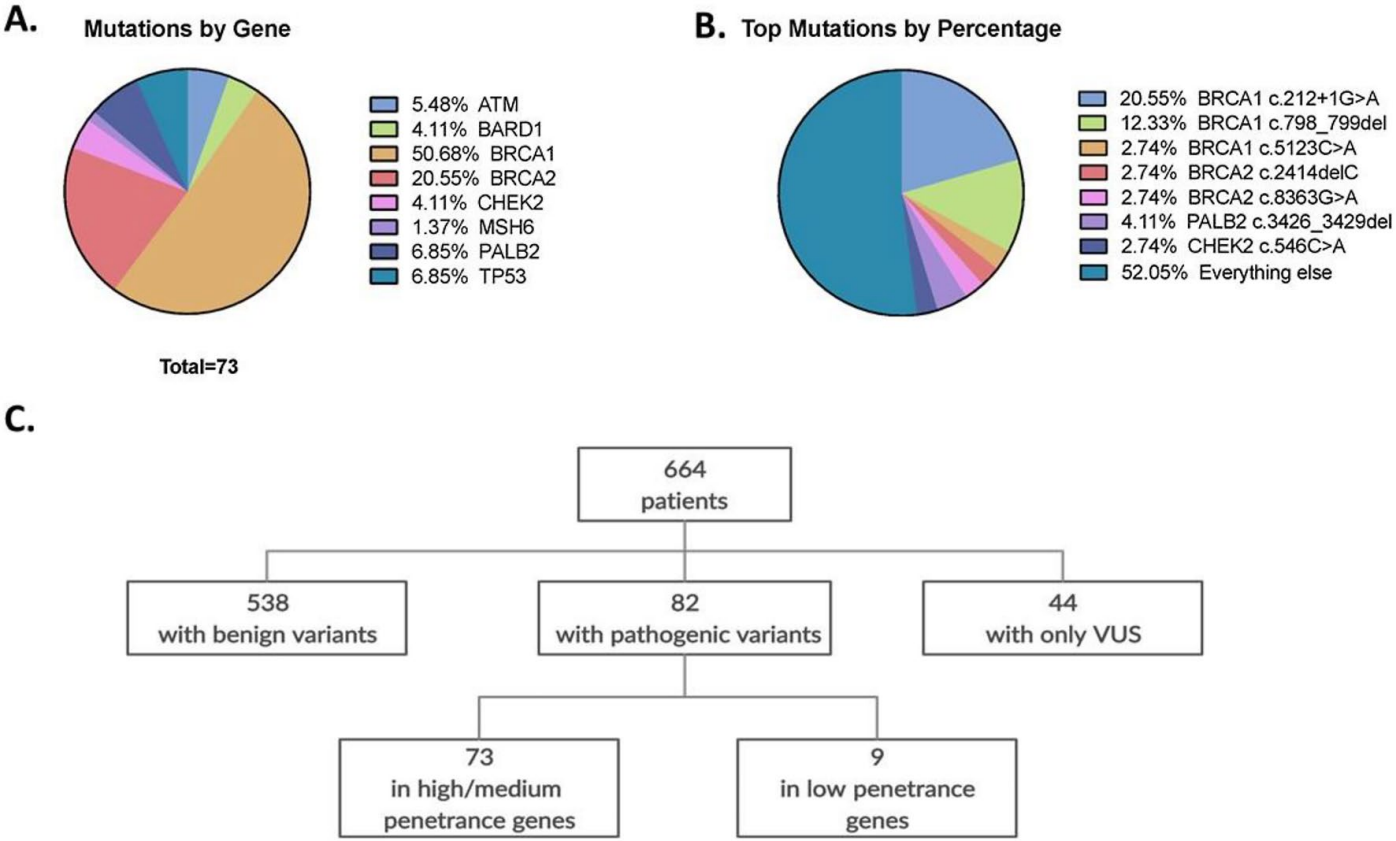
Pathogenic mutations in high and moderate penetrance genes. **A** Shown is the percentage of all pathogenic mutations in moderate and high penetrance genes, by gene or **B** by recurrent mutation. **C** The groupings of patients are shown by variant type

We identified 45 independent pathogenic variants in *ATM*, *BARD1*, *BRCA1*, *BRCA2*, *CHEK2*, *MSH6*, *PALB2*, and *TP53*, in 73 subjects. In addition we identified 9 rare pathogenic variants in the low/unknown-penetrance genes *AXIN2, FH, MLH1, MUTYH, NF1, and SDHB*. Mutations in *BRCA1* accounted for 51% of non-rare pathogenic variants (37/73), followed by *BRCA2* (21%; 15/73), *PALB2* and *TP53* (6.9%; 5/73) each, *ATM* (5.5%; 4/73), and *BARD1* and *CHEK2* (4.1%; 3/73) each (Fig. 1A, B).

### Association of mutations with age at diagnosis and family history

Of the 664 patients, 538 have no variant or “benign” variants (variants confirmed to be non-pathogenic), 82 have pathogenic variants, and 44 have variants of uncertain significance (VUS) (Fig. 1C, Table 1). Clinical characteristics of patients with pathogenic mutations in high or moderate penetrance genes are compared to all other patients. Patients with pathogenic mutations in high or moderate penetrance genes had a significantly earlier age at diagnosis (median, 43 years; IQR 37–52) compared to all others (median, 49 years; IQR 41–61) (*P* < 0.01) (Table 1). Also, 33% of patients with pathogenic variants in high or moderate penetrance genes reported a family member with breast cancer compared to 15% of all other patients (*P* < 0.001). Patients with non-pathogenic mutations were more likely to have had menopause before diagnosis than patients with pathogenic mutations. Although factors such as the number of pregnancies and breastfeeding have been associated with breast cancer risk [15, 16], we found no significant difference in these variables between carriers of pathogenic variants vs. non-carriers. This suggests that in this sample of patients from Guatemala, women with hereditary breast cancer do not have a different reproductive risk profile as compared to those with sporadic breast cancer.

### Recurrent mutations

We identified eight recurrent pathogenic mutations including three in *BRCA1* (c.799delT, c.212 + 1G > A, c.5123C > A), two in *BRCA2* (c.8363G > A, c.2414delC), one in *CHEK2* (c.546C > A), one in *PALB2* (c.3426_3429del), and one in *MUTYH* (c.1218_1219dup) (Fig. 1B). The *BRCA1* variants c.212 + 1G > A (rs80358042) and c.799delT (rs80357724) occurred most frequently, accounting for 19% and 12% of all pathogenic mutations, respectively (Fig. 1B). The c.212 + 1G > A (rs80358042) is a splice site mutation, whereas c.798_799del (rs80357724) is a frameshift variant; therefore, both mutations result in premature stop codons.

To analyze the impact of the recurrent pathogenic mutations, we compared them as a group to the rare pathogenic mutations. No significant differences in clinical presentation could be detected between those with a recurrent and a non-recurrent pathogenic mutation. (Supplemental Table 4).

### Variants of uncertain significance

Fifty-four variants were identified in high penetrance breast cancer genes with no definitive interpretation in ClinVar or Varsome. These 54 VUS were in 53 patients, with one patient containing 2 VUS and nine patients also carrying pathogenic variants, for a total of 44 (6.6%) unique patients with a VUS only. Thirteen VUS were in *BRCA1*, thirty in *BRCA2*, seven in *PALB2*, and two in *PTEN* and *TP53.* A total of 6 were recurrent mutations and 45 were unique. The percentage of independent VUS in each high penetrance gene is 29% (2/7) in *TP53*, 43% (12/28) in *BRCA1*, 63% (5/8) in *PALB2*, 65% (24/37) in *BRCA2*, and 100% (2/2) in *PTEN* (Supplemental Table 5).

In addition to the VUS, we identified pathogenic variants in genes proposed but not demonstrated to be involved in inherited breast cancer (*AXIN2*, *FH*, *MLH1, MSH2, MUTYH, NF1*, and *SDHB)*. We did not include these variants in our analyses, as they are unlikely to be directly linked to breast cancer. We also observed rare, non-coding variants in high penetrance genes (*BRCA1, BRCA2, PALB2, TP53*). None of these variants appeared to be pathogenic from our additional risk assessment using Align-GVGD. However, these variants may affect splicing or protein function (Supplemental Table 3).

### NCCN guidelines for genetic testing

The National Comprehensive Cancer Network (NCCN) guidelines are recognized as the standard for clinical decision-making and consider risk factors such as age and family history to recommend genetic testing. We determined the utility of the NCCN guidelines in identifying Guatemalan patients with pathogenic mutations. We found that 77% of patients with pathogenic variants in high or moderate penetrance breast cancer genes met NCCN criteria for genetic screening compared to 59% of all others (Table 1). Therefore, these guidelines will be useful for clinical genetic decision-making.

## Discussion

To estimate the frequency and spectrum of germline mutations in the Guatemalan population, we studied 664 unselected breast cancer patients from two large hospitals in Guatemala. We found 11% (73/664) carry pathogenic variants in high and moderate penetrance breast cancer susceptibility genes. Those women with pathogenic mutations have an earlier age of onset, are more likely to have premenopausal breast cancer, and a higher proportion have a family history of breast cancer. These data provide essential information to the development of genetic screening and treatment programs for Guatemala. Given the extensive shared genetic ancestry, these data are also relevant to women in Mexico and Central America and US Hispanic women.

The Guatemalan women included in our study have a higher ratio of deleterious *BRCA1*/*BRCA2* mutations (2.8) than published data on the US Hispanic women or other Latin American populations [3–6]. The higher *BRCA1* mutation rate is attributable to the high prevalence of the c.212 + 1G > A and c.799delT mutations. These two mutations are not known to be frequent in the United States and other Latin American countries, suggesting that they are founder mutations in the Guatemalan population [3, 6, 8]. A previous study of 222 patients in four Latin American countries (Argentina, Colombia, Guatemala, and Mexico) described a prevalence of pathogenic variants at 17% (38/222); however, if only high and moderate penetrance breast cancer genes are included, that drops to 13% (29/222) overall and 11% (2/19) in Guatemala [6]. The BRCA mutation prevalence among Latinas in the US is 1.2% to 4.9% in patients with breast cancer. A separate study of 1054 BRCA-negative, high-risk Hispanic women found that 4.5% carried a pathogenic variant in other cancer susceptibility genes [8, 16].

Due to the unique population history of Latin American countries and Hispanic populations, several recurrent mutations have been observed, including a deletion of exons 9–12 in *BRCA1* observed in Mexico and Mexican Americans, and *BRCA2* E1308X in Puerto Rico [3, 5, 7]. One recurrent mutation that has been documented in several populations across Latin America and U.S. Hispanics is the Jewish founder mutation *BRCA1 185delAG* [4, 17]. However, this mutation was not seen in the Guatemalan population. The six recurrent mutations discovered (*BRCA1* c.212 + 1G > A, *BRCA1* c.799delT, *BRCA1* c.5123C > A, *BRCA2* c.8363G > A, *BRCA2* c.2414delC, and *PALB2* c.3426_3429del) seem to be uniquely recurrent within Guatemala. With increasing intraregional migration rates, particularly to Mexico and the United States, geneticists in Central and North America should look for these mutations when screening Guatemalan patients. Particular attention should be given to *BRCA1* mutations c.212 + 1G > A and *BRCA1* c.799delT, which were the most prevalent within our study population. These two mutations were also seen once in the 19 Guatemalan patients included in Oliver et al. [6].

The high percentage of variants in high and moderate penetrance genes classified as VUS suggests that the current online databases are still underpowered, especially for Latin American samples. As the second most commonly affected gene in hereditary breast and ovarian cancer, *BRCA2* especially warrants further study. One variant, *BRCA2* p.Leu2962_Asp2983del is notable for being an in-frame deletion of 22 amino acids, classified as a VUS in ClinVar. We identified this deletion in 4 Guatemalan patients who do not have early-onset disease nor a family history of breast cancer, suggesting this is either a benign or hypermorphic allele. Additionally, several pathogenic mutations were detected in genes with low or unknown penetrance for breast cancer. Further research is needed to clarify whether these VUS and rare pathogenic mutations are clinically actionable.

Consistent with current knowledge, patients with pathogenic mutations in known susceptibility genes had a lower averge age at diagnosis than those with benign mutations. A family history of breast cancer was also elevated in patients with pathogenic mutations. At an average of 51 years, the age of breast cancer diagnosis in our sample of Guatemalan patients was lower than the U.S. average of 62 years [18]. Although this is likely due to the younger age structure of the population, it suggests that women from Guatemala may benefit from starting annual mammograms at an earlier age. However, this would require a more significant outreach effort to communities outside of Guatemala City and Mayan-speaking communities.

Amerindian ancestry is associated with a lower incidence of breast cancer, partly due to a protective allele at rs140068132 common in Mexican, Central, and South American populations [19]. Guatemalans have a high percentage of Amerindian ancestry, and 30–40% speak one of the Mayan languages as their primary language. Our study found a low rate of women (< 1%) speaking a Maya language, lower than the 7% of women in a parallel survey of cervical cancer carried out in the same clinic at INCAN [20]. A case–control study of breast cancer in Central America is warranted as to gain additional information from this population on the role of germline variants in breast cancer risk.

Finally, our data show that the NCCN guidelines for breast cancer genetic testing are reasonably effective for Guatemala. A total of 77% of patients with pathogenic mutations in high or moderate penetrance genes would have qualified for testing. However, with the diversity of breast cancer patients, these guidelines may need to be adjusted on a regional or national basis.

This is the most extensive genetic study of Central American breast cancer, to the best of our knowledge. Our research has several limitations: the populations were hospital-based case samples in the capital city and possibly skewed for women of higher socioeconomic status, women with more advanced disease, and not representative of rural populations. We documented that indigenous women whose primary language is not Spanish are under-represented in our sample. This finding may be due to under-representation in indigenous women seeking treatment or learning about and enrolling in our study [21]. Mestizo women are more likely to have received education about breast cancer and seek screening at an earlier age. Another limitation is that cancer history is self-reported and not verified by medical records. Finally, there is a lack of hormone receptor status and histology data that could lend insight into the specific breast cancer subtypes present in this patient population, as well as biopsy confirmation of invasive disease for the INCAN patients.

## Supplementary Information

Below is the link to the electronic supplementary material.

Supplementary file1 (XLSX 56 kb)

## Data Availability

DNA sequencing data will be made available to qualified researchers through dbGAP (https://www.ncbi.nlm.nih.gov/gap/).

## Abbreviations

IRB: Institutional review board
SES: Socioeconomic status
RR: Relative risk
NCCN: National Comprehensive Cancer Network
VUS: Variant of unknown significance
